# Prevention of thrombotic disorders by antithrombotic diet and exercise: evidence by using global thrombosis tests

**DOI:** 10.4155/fsoa-2017-0104

**Published:** 2018-01-24

**Authors:** Junichiro Yamamoto, Yoshinobu Ijiri, Hideo Ikarugi, Kazunori Otsui, Nobutaka Inoue, Kjell S Sakariassen

**Affiliations:** 1 Kobe Gakuin University, Kobe 651–2180, Japan; 2 Faculty of Health & Nutrition, Osaka Shoin Women's University, Osaka 577–8550, Japan; 3 School of Economics, University of Hyogo, Kobe 651–2197, Japan; 4 Clinical Department of General Internal Medicine, Kobe University Hospital, Kobe 650–0017, Japan; 5 Department of Cardiovascular Medicine, Kobe Rosai Hospital, Kobe 651–0053, Japan; 6 KellSa s.a.s. Str. Campo e Zampo 12, I-13900 Biella, BI, Italy

**Keywords:** antithrombotic vegetables, cardiovascular disease, exercise paradox, fibrinolysis, global parallel-plate thrombosis test, global thrombosis test, platelet aggregation, shear rate, shear stress, stroke

## Abstract

Prevention of thrombotic disorders has priority over treatment. There are only two pathologically relevant tests which are suitable for measuring the overall thrombotic status both in experimental conditions and in humans. The Global Thrombosis Test (GTT) and the Global Parallel-Plate Thrombosis Test can detect the pathologically relevant global thrombotic status. These tests have been successfully used for monitoring the effect of antithrombotic drugs and for developing novel antithrombotic agents. By using GTT, varieties of fruits, vegetables, and regular physical exercise have been tested for the effect on global thrombotic status. This review discusses the published evidence for the benefit of diet of selected fruit and vegetable varieties and doing regular physical exercise on improving thrombotic status. Future clinical trials monitored by GTT or Global Parallel-Plate Thrombosis Test could decide on the effectiveness of an experimentally proven antithrombotic diet with regular physical exercise in the prevention of thrombotic diseases.

## Lay abstract

Prevention of deadly diseases like heart attack or stroke is more effective than treating patients with already progressed disease. Epidemiologic studies raised the possibility that eating healthy food and doing physical exercise may prevent thrombotic diseases. To create an ‘antithrombotic diet’, fruits and vegetables should be selected and the benefit of diet and exercise should be monitored in people. We found that the Global Thrombosis Tests are useful for both selection of antithrombotic diet components and monitoring thrombotic status of individuals.

## Arterial thrombosis/thrombolysis *in vivo* tests in experimental animals

**Figure 1.**
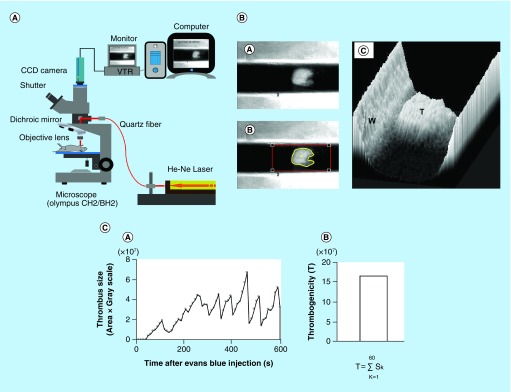
**Measurement of thrombotic status in rodents (*in vivo*).** He-Ne laser-induced thrombosis system **(A)**. Measurement of thrombus size in mouse carotid artery **(B)**. Thrombus formed in carotid artery **(A)**; thrombus delineated by software **(B)**; thrombus formed by software **(C)**. Change in thrombus size over 600 s and calculation of the index of thrombotic status **(C)**. Consecutive thrombus size over time **(A)**; an index of thrombogenicity **(B)**. He-Ne: Helium-Neon; T: Thrombus formed by software; W: Vessel wall.

Techniques using laser irradiation to induce vascular injury and subsequent platelet-rich thrombus formation were established in the early 1970s [1–10]. Helium-Neon (He-Ne) laser-induced thrombosis *in vivo* model, which selectively damaged the vascular endothelium has been used extensively in thrombosis research [11–16]. Yamamoto *et al*. found a good correlation between a shear-induced *ex vivo* global thrombosis test and the ‘gold standard’ He-Ne laser-induced thrombosis *in vivo* test [17]. The He-Ne laser-induced *in vivo* thrombosis test allowed the assessment not just the formation and growth of thrombus but also its fragmentation, embolism and lysis [18–23]. Irradiating the carotid artery of a mouse with He-Ne laser, real-time measurement of thrombus size and its changes over time is shown in Figure 1.

### Technical details of He-Ne laser thrombosis model

Evans blue was injected into a rat anesthetized with sodium pentobarbital. An intestinal loop was spread out flat on a self-constructed object stage, which was mounted on the adjustable plate of an Olympus microscope. An O-ring was placed on the mesentery to stop the movement of the vessel. Selected mesenteric arterioles and venules were irradiated with a focused He-Ne laser beam of 15 μm diameter. A single 5 s irradiation was repeated every 30 s until an occlusive thrombus was formed. The number of laser irradiations is necessary to induce fully occlusive thrombus formation was counted. The antithrombotic effect was assessed by the increase of the number of irradiations necessary to occlusion [11]. Similarly, thrombus was formed in the carotid artery of mice [12]. An indwelling catheter was inserted into the left femoral artery and the left carotid artery was exposed. Having transferred the mouse onto a microscope stage, laser beam (200 μm in diameter at the focal plane) was targeted onto the center of the exposed carotid artery. Thrombus formation was monitored under fiberoptic epi-illumination through a microscope with an attached CCD camera and recorded with a video recorder. An image of the thrombus was taken every 10 s over 600 s and the thrombus size was analyzed by Analyst software. In brief, the lumen of the laser-targeted vessel was fitted into an optical frame, the grayscale threshold level was set to delineate the thrombus, and the thrombus area was measured. The thrombus size was calculated by multiplication of the area and grayscale values. During the irradiations, the size of thrombus mass changed due to growth and embolization, but complete occlusion did not occur. A total sum of 60 individual images taken over 600 s were analyzed to calculate the index of thrombogenicity [13].

## Platelet-rich thrombus formation *ex vivo* in native blood under flow conditions

Plasma fibrinolytic activity can be assessed *in vitro* or *in vivo*, by measuring various biomarkers. Measurement of thrombolysis under flow revealed the important contribution of platelets not just to the formation but also to lysis of a thrombus [24–32].

## Arterial & venous thrombus formation triggered by arterial subendothelium, collagen or tissue factor/phospholipids in perfusion chambers under normal & atherosclerotic native blood flow conditions

New frontier research was developed by HR Baumgartner and colleagues and by KS Sakariassen and colleagues by the developments and validations of thrombus formation triggered by thrombotic surfaces in annular and parallel-plate blood perfusion chambers [33–35].

**Figure 2.**
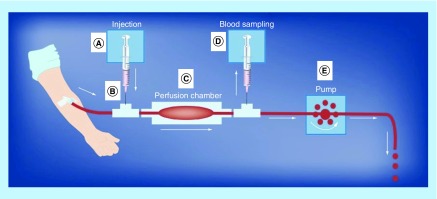
**The global parallel-plate blood perfusion chambers test.** The various components of the blood perfusions system are as follows. **(A)** pump for injection of test agents; **(B)** mixing chamber device for test agents in the flowing blood stream; **(C)** parallel-plate perfusion chamber with thrombogenic surface; **(D)** pump for drawing of postchamber blood samples for bioanalysis; **(E)** pump drawing blood from vein through perfusion chamber. Reproduced with permission from [37].

Important features of this technique were the use of native blood (nonanticoagulated blood) at venous, arterial and atherosclerotic blood flow conditions. The blood was drawn from an antecubital vein through a 19-gauge syringe at 10 ml/min by a peristaltic roller pump placed distal to the perfusion chamber as shown in Figure 2. The respective blood wall shear rates in the parallel-plate perfusion chambers were 100 s^-1^ (venous blood flow), 650 s^-1^ (averaged-sized arteries), 2600 s^-1^ (moderate arterial stenosis – stenosis occlusion of 60%), 10,500 s^-1^ (severe arterial stenosis – stenosis occlusion of 80%) and 32,000 s^-1^ (very severe stenosis occlusion – stenosis occlusion of 89%). The thrombogenic surfaces of the perfusion chambers consist of either human vascular subendothelium, collagen or tissue factor/phospholipids. Parameters of thrombus formation measured by morphology are platelet surface adhesion, size of thrombi and fibrin deposition. Complementary methods to measure fibrin and platelet deposition by immunological methods were developed as well. Biomarkers of platelet activation, coagulation and fibrinolysis can be measured from blood samples collected downstream to the site of thrombus formation during the blood perfusion period. A corresponding miniature parallel-plate perfusion chamber was also developed where thrombus formation is studied simultaneously at three different shear conditions at a blood flow rate of 1 ml/min [36].

The parallel-plate perfusion chambers have been validated with a large number of studies including patients with bleeding disorders and patients on antithrombotic agents, both platelet inhibitors and coagulation inhibitors [38,39]. Also, combination therapies with platelet inhibitors and coagulation inhibitors were included.

## Arterial thrombosis test (POC) performed from native blood under pathologically relevant flow conditions

**Figure 3.**
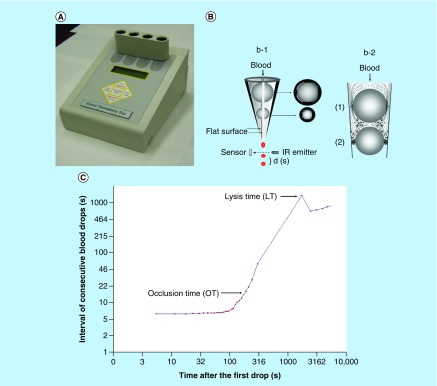
**GTTmeasurement using native blood.** GTT instrument **(A)** and principle of the measurement **(B)**. Platelets in native blood are activated by high shear forces in the upper gaps (1). Fibrin stabilized thrombi formed between the two bearing balls occlude the lower gaps (2). Real time recording of GTT measurement **(C)**. IR: Infra red; GTT: Global thrombosis test; LT: Lysis time, time from the start of measurement until the first blood drop detected after OT + 200 s stabilization; OT: Time between two consecutive blood drops exceeding the default (15 s).

Unique features of the point-of-care (POC) instrument developed by Kovacs *et al*., are the testing native blood and initiating thrombus formation solely by pathologically relevant high shear stress [40–45]. The POC shear-induced test drives the tested native blood to flow through narrow gaps and thrombi formed in the poststenotic area are captured in a distal second gap, thus arresting the flow in the system. The technique measures the time of occlusive thrombus formation and the subsequent endogenous lysis of the occlusion. The principle of Global Thrombosis Test (GTT) is shown in Figure 3.

The measurement starts by withdrawal of blood sample from the antecubital vein using a 21G needle with a butterfly cannula. To minimize platelet activation during blood sampling, the double syringe technique was used in each subject. The first 3 ml blood was used for routine blood tests, and the subsequent 4.0 ml was used for GTT measurement [44,45]. Syringe with the blood sample was inserted into the disposable GTT test tube and the measurements started within 15 s from withdrawing the blood. During the measurement blood flows under gravity through small gaps, in which platelets are activated by the initial high shear rate (12,000–14,000 s^-1^). Formation of fibrin-stabilized thrombus and its lysis are detected.

## High shear-induced *ex vivo* thrombosis/fibrinolysis test of native blood sample: comparison with conventional tests

GTT proved to be sensitive in detecting small differences in thrombotic and fibrinolytic activities between age, gender, smoker or nonsmoker people [46–48], in metabolic syndrome [49] and in stroke patients [50]. In these studies, GTT surpassed the sensitivity of routine coagulation tests like the prothrombin time test and activated partial thromboplastin time test especially in monitoring oral anticoagulants [51]. GTT detected hyper thrombotic status after overwork, which could not be detected by conventional coagulation tests [52].

The benefit of using GTT in various clinical conditions (myocardial infarction, atrial fibrillation, monitoring dual antiplatelet or oral thrombin inhibitor medications after coronary angioplasty), has been documented in several trials involving large number of patients [53–58].

Reproducibility of GTT has been tested and published. If blood sampling is performed by a trained operator, the reproducibility of both thrombotic (OT) and thrombolytic activity (LT) is good (the intra-assay CV was OT = 10% and for LT = 6% and the inter-assay CV was OT = 8% and for LT = 9%) and allows credible statistical analysis of the findings [55].

## Prevention of thrombotic disorders by long-term antithrombotic diet of fruits & vegetables & doing physical exercise

It is widely believed that intake of fruits, vegetables and physical exercise is beneficial to thrombotic status especially in those who are at risk of thrombotic diseases. In many countries recommendation for an antithrombotic diet and adequate physical exercise has been proposed to the relevant health authorities (Table 1) [59–66].

**Table 1.** **Recommendation for consumption of fruits, vegetables and doing regular physical exercise.**

**Country**	**Vegetables**	**Fruits**	**Ref.**

	**Target for daily intake of vegetables**	**Target for daily intake of fruits**	
Japan	350 g	More than 100 g	[65]

NL	At least 200 g	At least 200 g	[59]

UK	At least five portions (400 g) of a variety of fruit and vegetables		[60]

USA	2.5 cup (approximately 600 g) for 2000-calorie level pattern	Two cups (approximately 480 g) for 2000-calorie level pattern	[61]

**Physical exercise**			

Japan	Adults (aged 20–64 years): male 9000 steps, female 8500 steps per day; Older adults (65 years and older): male 7000 steps, female 6000 steps per day		[66]

NL	Adults (aged 18–54 years): at least 30 min of moderate- to vigorous-intensity physical activity with moderate defined as 4.0–6.4 METs and vigorous defined as ≥6.5 METs on a minimum of 5 days per week. Older adults (55 years and older): at least 30 min of moderate defined as 3.0–4.9 METs and vigorous defined as ≥5.0 METs on a minimum of 5 days per week. For inactive people: any additional amount of any type of exercise is considered useful, regardless of intensity, duration and frequency		[62]

UK	Adults (aged 19–64 years): at least 150 min of moderate-intensity aerobic activity a week plus muscle strengthening activities on two days or more of the week; or 75 min of vigorous intensity aerobic activity plus muscle strengthening activities on 2 days or more of the week; or a combination of moderate and vigorous aerobic activity every week. Older adults (65 years and older): at least 2 days a week, try to take part in types of activities such as cycling, Tai chi, yoga or stretching exercises. And remember, it is never too late to turn over a new leaf and become more active – just go easy to start with and build up your fitness gradually. If 30 min all in one go sounds a bit too much to start with, do not worry – you can make up the daily 30 min by adding together shorter bouts of activity, of at least 10 min each		[63]

US	Adults (aged 18–64 years): at least 150 min a week of moderate-intensity, or 75 min a week of vigorous-intensity aerobic physical activity, or an equivalent combination of moderate-and vigorous-intensity aerobic activity. Aerobic activity should be performed in episodes of at least 10 min, and preferably, it should be spread throughout the week. Adults should also include muscle-strengthening activities that involve all major muscle groups on 2 or more days a week. Older adults (65 years and older): when older adults cannot meet the adult guidelines, they should be physically active as their abilities and conditions will allow. Older adults should do exercises that maintain or improve balance if they are at risk of falling		[64]

MET: Metabolic equivalent of task.

### Effect of fruits & vegetables on thrombotic status

Quality and quantity of daily intake of fruits and vegetables shows great differences in various countries. Large epidemiological study in the USA found that the risk of coronary heart disease in adults depends on the consumption of healthful and unhealthful plants [67]. In this study fruits, vegetables and other processed diets were classified as ‘healthy’ (raisins, grapes, prunes, bananas, cantaloupe, watermelon, fresh apples or pears, oranges, grapefruit, strawberries, blueberries, peaches or apricots or plums, tomatoes, tomato juice, tomato sauce, broccoli, cabbage, cauliflower, Brussels sprouts, carrots, mixed vegetables, yellow or winter squash, eggplant or zucchini, yams or sweet potatoes, spinach cooked, spinach raw, kale or mustard orchard greens, iceberg or head lettuce, romaine or leaf lettuce, celery, mushrooms, beets, alfalfa sprouts, garlic, corn, nuts, peanut butter, string beans, tofu or soybeans, beans or lentils, peas or lima beans, vegetable oil used for cooking, tea, coffee, decaffeinated coffee) and ‘less healthy’ (apple cider or juice, orange, grapefruit and other fruit juice, white rice, baked or mashed potatoes, potato or corn chips etc).

By testing native blood using the shear-induced GTT, Yamamoto *et al*. found that antithrombotic activity of fruits and vegetables classified as ‘healthy’ is still depend on the tested varieties. Accordingly, varieties of the same fruit or vegetable can be antithrombotic, prothrombotic or without effect on thrombotic status [68–74]. The antithrombotic varieties demonstrated in animal experiments were effective in short and long-term intake by humans [75,76]. Thus, selection of varieties of fruits and vegetables for antithrombotic effect by GTT is a promising approach to developing antithrombotic diet for the prevention of thrombotic disorders such as cardiovascular disease and stroke. Large scale studies and trials are needed to verify this claim.

### Physical exercise & exercise paradox

Epidemiological and clinical studies suggested that regular exercise is an efficient way to prevent arterial thrombotic diseases [77–81]. The recommended ‘government guidelines’ is shown in Table 1. Despite such recommendation, the benefit of exercise is still not generally accepted, debated and objections are referred as ‘exercise paradox’ or ‘double-edged sword in exercise’ [82–86]. Ikarugi and Yamamoto proposed that regular assessment of thrombotic status using GTT may be helpful in individualizing the effect of exercise in people at risk [87].

## Limitations of tests using native blood

The requirement of starting the tests within 15–20 s after withdrawal of blood samples mandates experienced operator or phlebotomist, and organization that the instrument should be near to the tested person. It is the latter which is most difficult to ensure, as in the present clinical practice blood samples are taken, stored and tested later in different laboratories.

## Conclusion & future perspective

The concept of preventing arterial thrombotic disorders by long-term intake of an experimentally proven antithrombotic diet and monitored regular physical exercise is challenging and intriguing. The key to success is finding test(s) capable of monitoring thrombotic status under pathologically relevant conditions. In contrast to common platelet function or coagulation tests currently in use, the shear-induced global thrombosis and thrombolysis test and the global parallel-plate thrombosis test performed with native (nonanticoagulated) blood are suitable for screening fruit and vegetable varieties for antithrombotic effect. The use of these tests under experimental conditions can result in establishing an antithrombotic diet. Having found the pathologically relevant tools of testing and monitoring thrombotic status of individuals, future work should be focused on organizing large scale and monitored trials involving healthy people, and those who are at risk of atherothrombotic diseases and cardiovascular events. The outcome of such trials may justify the everyday consumption of an antithrombotic diet with regular physical exercise, and this would be a simple and economical way of prevention of arterial thrombotic diseases.

**Executive summary**

- Interest in prevention of thrombotic disorders by antithrombotic vegetable varieties and physical exercise is increasing. Pathologically relevant point-of-care global tests for the assessment of global thrombotic status are badly needed.
- The Global Thrombosis Test enables the simultaneous measurement of platelet reactivity to high shear stress, platelet procoagulant and endogenous fibrinolytic activity. This technique proved to be suitable for screening fruits and vegetables for antithrombotic effect, making possible to establish an antithrombotic diet and also to individualize the need of physical exercise in people at risk of thrombotic events. The Global Parallel-Plate Thrombosis Test enables also the simultaneous measurements of platelet activation, coagulation and fibrinolytic activities during thrombus formation.
- Global Thrombosis Test-monitored or Global Parallel-Plate Thrombosis Test-monitored large scale trials are needed to verify the beneficial effect of an antithrombotic diet with regular physical exercise in the prevention of arterial thrombotic events.
